# Supplementary material for: Bidirectional redistribution of actomyosin drives epithelial invagination in ascidian siphon tube morphogenesis
Source: eLife. 2026 Oct 1;14:RP108588. doi: 10.7554/eLife.108588 (PMC13630385; doi:10.7554/eLife.108588)
Supplement: Peer review history

# Editor report: eLife Assessment

**DOI:** 10.7554/eLife.108588.4.sa0

**Authors:** Deborah Yelon

**Affiliations:** University of California, San Diego United States

**Keywords:** Convincing, Important

This **important** study uses a combination of experimental and modeling approaches to investigate the role of actomyosin in epithelial invagination during Ciona siphon tube morphogenesis. Several types of **convincing** quantitative analyses and modeling approaches are presented that support a model in which bidirectional relocation of actomyosin drives invagination. Since epithelial invagination contributes to the morphogenesis of many developing organs, this work has the potential to appeal to both cell biologists and developmental biologists.

# Referee report: Reviewer #2 (Public review)

**DOI:** 10.7554/eLife.108588.4.sa1

Summary:

The authors propose that bidirectional redistribution of actomyosin drives tissue invagination in Ciona siphon tube formation. They suggest a two-stage model where actomyosin first accumulates apically to drive a slow initial invagination, followed by redistribution to lateral domains to accelerate the invagination process through cell shortening. They have shown that actomyosin activity is important for invagination - modulation of myosin activity through expression of myosin mutants altered the timing and speed of invagination; furthermore, optogenetic inhibition of myosin during the transition of the slow and fast stages disrupted invagination. The authors further developed a vertex model to validate the relationship between contractile force distribution and epithelial invagination.

Strengths:

(1) The authors employed various techniques to address the research question, including optogenetics, use of MRLC mutants, and vertex modelling.

(2) The authors provide quantitative analyses for a substantial portion of their imaging data, including cell and tissue geometry parameters as well as actin and myosin distributions. The sample sizes used in these analyses appear appropriate.

(3) The authors combined experimental measurements with computer modeling to test the proposed mechanical models, which represents a strength of the study. It provides a framework to explore the mechanical principles underlying the observed morphogenesis.

Comments on revised version.

The authors have adequately addressed my previous concerns regarding the optogenetic experiments, and the addition of the new modeling analysis further strengthens the study.

# Author comment: Author response

**DOI:** 10.7554/eLife.108588.4.sa2

**Authors:** Jinghan Qiao, Pengyu Yu, Hongzhe Peng, Wenjie Shi, Bo Li, Bo Dong

**Affiliations:** Ocean University of China Qingdao China; Tsinghua University Beijing China; Ocean University of China Qingdao China; Ocean University of China Qingdao China; Tsinghua University Tsinghua China; Ocean University of China Qingdao China

The following is the authors’ response to the previous reviews.

**Public Reviews:**

**Reviewer #1 (Public review):**

This is an extensively revised version of a previously submitted manuscript that, as detailed in their 20-page response to the first reviews, satisfactorily addresses the reviewers' comments. In particular, the revised manuscript makes it much clearer how this work fits into and advances the field. The added experiments strengthen the rigor of the manuscript as well. Overall, this paper is ready to go.

We thank the reviewer for the positive evaluation and for recognizing the improvements made in our revised manuscript.

**Reviewer #2 (Public review):**

The revised manuscript has been substantially improved. The authors have addressed many of my previous concerns through the addition of new data, analyses, and discussion. The characterization of epithelial folding in the ascidian Ciona provides valuable insight into a comparatively less explored morphogenetic system, and the imaging and quantitative analyses are overall compelling. That said, a few important points remain to be addressed.

We thank the reviewer for the positive assessment and for acknowledging the improvements made in our revised manuscript. We are also grateful for the reviewer’s continued constructive suggestions.

One remaining issue concerns the mechanistic novelty of the actomyosin redistribution described in this study. The authors emphasize that the key novelty lies in the stepwise translocation of actomyosin from the lateral membrane to the apical domain during the initial stage (apical constriction), followed by redistribution from the apical domain back to the lateral domain during the accelerated stage (invagination). I agree that the dynamic redistribution itself is potentially interesting and may represent an underexplored aspect of epithelial morphogenesis. However, as I discussed in my previous review comments, from a mechanics perspective, the role of apical actomyosin in driving apical constriction and of lateral actomyosin in contributing to tissue folding/invagination have already been demonstrated in multiple systems, although to varying extents depending on the model. Therefore, while the current study convincingly documents a distinct spatiotemporal sequence of actomyosin localization in Ciona atrial siphon tube formation, it could be clarified further to what extent this work advances new mechanical principles underlying epithelial folding, as opposed to revealing a variation in the deployment of previously described force-generating modules.

Importantly, I think the manuscript has the potential to provide deeper conceptual insight if the authors more explicitly consider the significance of the "redistribution" process itself. Redistribution does not only involve the appearance of actomyosin at a new membrane domain; it also necessarily involves its disappearance from the previous domain. The latter aspect has, in my view, been much less explored in the literature. For example: Is the removal of lateral actomyosin during the early phase important for efficient apical constriction? Conversely, is the reduction of apical actomyosin during the later accelerated phase important for proper invagination mechanics? These questions are particularly interesting because they address whether redistribution between domains serves an active mechanical regulatory role, rather than focusing on the role of force-generating actomyosin at a given location.

I acknowledge that addressing these questions experimentally could be technically challenging. One potentially powerful way to address this would be through the revised computational model. For example, the authors could test whether tissue folding is altered when actomyosin is allowed to accumulate at a new domain without being concomitantly depleted from the original domain. Such analyses could help distinguish whether redistribution itself has functional mechanical importance, rather than merely reflecting sequential recruitment to different cellular regions. In my opinion, incorporating this aspect would substantially strengthen the conceptual and mechanistic novelty of the study.

We thank the reviewer for raising this important point. We agree that the mechanical significance of actomyosin redistribution, beyond the individual roles of apical and lateral contractility, deserves further clarification. In our study, quantitative analysis of F-actin dynamics revealed a bidirectional reorganization of the actomyosin network during siphon morphogenesis: the increase of F-actin intensity in one domain was accompanied by its reduction in the other domains during both the initial and accelerated invagination stages. These observations suggest that actomyosin redistribution may represent an active mechanical regulatory process rather than merely sequential recruitment to different cellular domains. Following the reviewer’s suggestion, we used the computational model developed in this study to examine its functional significance.

Specifically, we modified the temporal dynamics of actomyosin activity by maintaining apical contractility during the accelerated stage or by prematurely enhancing lateral contractility during the initial stage in simulations. We found that sustained apical tension during the accelerated stage primarily induced stronger central cell elongation (Figure 6—figure supplement 1A-C), whereas elevating lateral actomyosin activity during the initial stage (14–16 hpf) suppressed central cell elongation and reduced the inward movement of surrounding cells toward the central region (Fig. 6—figure supplement 1D-F), resulting in earlier bending deformation with a flatter invaginating morphology (Fig. 6—figure supplement 1F). Although both perturbations eventually converged to similar final shapes, likely because they reached similar final actomyosin distributions, their distinct morphogenetic trajectories demonstrate the mechanical importance of the temporal sequence of actomyosin redistribution. These results further clarify the significance of the previously identified apico-basal tension imbalance and lateral contraction by revealing how their sequential activation coordinates tissue deformation. The initial dominance of apical contractility, together with limited lateral contraction, promotes cell elongation and convergence of the active region, whereas the subsequent shift toward lateral contractility facilitates cell shortening and deep tissue invagination.

Thus, our results highlight that the bidirectional redistribution of actomyosin is not merely a consequence of morphogenesis, but contributes to the dynamic regulation of epithelial folding. We have incorporated these new simulation results and analyses into the revised manuscript as Figure 6—figure supplement 1.

My other concern relates to the new optogenetic data presented in Figure 4-figure supplement 2. In the "Dark" samples, active myosin does not appear to be clearly enriched along the membrane, but instead seems relatively diffuse within the cytoplasm. This appears distinct from the images shown in Figure 2, where active myosin exhibits clear membrane enrichment. Could the authors provide top-view images for the samples shown in Figure 4-figure supplement 2? This would help clarify whether active myosin is indeed enriched along the apical membrane at 16 hpf and along the lateral membrane at 17 hpf in the "Dark" condition.

We thank the reviewer for this careful observation. We agree that the p-MLC signal in the "Dark" samples of the original Figure 4—figure supplement 2 appeared less membrane-enriched than in Figure 2. This was due to a technical adjustment: because the optogenetic system occupied the 568 nm channel, we have to switch the p-MLC signal from Alexa Fluor 568 anti-rabbit IgG to Alexa Fluor 647 anti-rabbit IgG, which yielded relatively weaker membrane signal under our imaging conditions. To address this, we have replaced the cross-sectional images with better-representative examples and added top-view images (Figure 4—figure supplement 2). These new panels clearly showed that active myosin was enriched at apical junctions (16 hpf) and lateral membranes (17 hpf) in the Dark condition, consistent with that in Figure 2. Since the Dark and Light groups were processed identically, the relative comparison remains valid.

In addition, the tissue morphology in the "17 hpf Light 1 hr" panel of Figure 4-figure supplement 2 appears noticeably different from that shown in Figure 4. Specifically, the apical side of the tissue in Figure 4 appears substantially more relaxed than in Figure 4-figure supplement 2. Based on the authors' interpretation of the optogenetic experiments, apical active myosin is not strongly affected by the treatment described in Figure 4. If so, one would expect apical constriction to remain largely intact. However, the more relaxed apical domain shown in Figure 4 seems to suggest that apical constriction may in fact be perturbed by the optogenetic manipulation. This apparent discrepancy complicates the interpretation of the experiment and seems somewhat inconsistent with the authors' main conclusion from this figure.

We thank the reviewer for this very careful and insightful observation. We agree that the tissue morphology in the "17 hpf Light 1 hr" panel of Figure 4—figure supplement 2 appears less relaxed than that in Figure 4B.

We acknowledge that optogenetic inhibition of myosin activity might indeed have a partial effect on activity of apical myosin, which can lead to apical relaxation after the initial constriction, as shown in the representative embryo in Figure 4B. However, because *Ciona* embryos are not always perfectly synchronized at 16 hpf (the time point when light illumination was initiated), individual embryos exhibit slight variations in the extent of apical constriction that has already been achieved during the initial stage (13.5–16.0 hpf). For embryos that had completed a relatively stronger apical constriction by 16 hpf, the apical domain can maintain its constricted morphology even after light exposure (Figure 4—figure supplement 2). Embryos with relatively weaker apical constriction at the time of light onset are more prone to exhibit apical relaxation upon optogenetic manipulation, as illustrated in Figure 4B. This developmental heterogeneity is the primary reason for the morphological variability observed between individual embryos in the optogenetic groups. Importantly, despite this morphological variability, the quantitative comparison of apical p-MLC intensity between the Light and Dark groups in Figure 4—figure supplement 2B showed no statistically significant difference (t-test, ns), which is consistent with the fact that during normal development, apical myosin activity naturally declines after 16 hpf (Figure 2B). In contrast, lateral p-MLC intensity was significantly reduced in the Light group compared to the Dark control (Figure 4—figure supplement 2B). This reduction in lateral contractility is the key factor responsible for the blockade of invagination progression.

We have revised the statements accordingly. Hopefully, these clarifications have adequately addressed the reviewer's concern.

**Reviewer #3 (Public review):**

Concerns raised in the initial submission were addressed in the revised manuscript.

We thank the reviewer for the encouraging feedback and for acknowledging our revisions.

**Recommendations for the authors:**

**Reviewer #1 (Recommendations for the authors):**

No further revisions suggested.

We thank the reviewer again for the positive assessment.

**Reviewer #2 (Recommendations for the authors):**

Here are several additional comments and suggestions in addition to the concerns described in the public review.

Line 86 - 88: the authors state "However, this transition is from apical to basolateral (Sherrard et al., 2010), rather than a bidirectional redistribution between apical and lateral domains." This statement feels somewhat out of context because the concept of "bidirectional redistribution" has not yet been introduced. It may fit more naturally in the Discussion section, after the relevant observations and interpretations have been fully presented.

We thank the reviewer for this suggestion. We have revised the sentence in the Introduction to avoid introducing the concept of "bidirectional redistribution" prematurely.

Line 92 - 95: the authors raised the questions of "Whether a bidirectional redistribution of actomyosin between apical and lateral domains operates as a core mechanism for sequential invagination, and whether lateral contractility is essential for the accelerated phase, remain unclear." These questions also feel somewhat out of context, for the same reason mentioned above.

We thank the reviewer for this suggestion. We have revised the text to avoid prematurely introducing the concept of "bidirectional redistribution" in the Introduction.

Line 162 - 163: The authors state that "This redistribution pattern was consistent with that of F-actin in the corresponding phases." This conclusion should be revised, as the reported increase in apical F-actin and reduction in lateral F-actin during the initial stage do not appear to reach statistical significance, which is different from that of active myosin.

We thank the reviewer for this careful observation. We agree that the F-actin changes during the initial stage did not reach statistical significance, unlike the active myosin data. We have revised the sentence to state that the myosin redistribution showed a similar trend to F-actin, while acknowledging the lack of statistical significance for F-actin.

Line 349 - 350: "and the invagination speed (represented by slopes of curves in Figure 5A) gradually slows down at later stages." It seems that Figure 5A should be Figure 5B.

Thank you for pointing out this typo. We have corrected it in the revised manuscript (now Figure 5C).

**Reviewer #3 (Recommendations for the authors):**

We appreciate the efforts made by the authors to address the questions and comments raised in the initial submission. The only remaining concerns are regarding grammar and spelling and a couple of minor errors.

(1) Lines 346-347, "These trends are consistent with the experimental mutant data (Figure 5A, B)."

This was confusing - was this meant to be Figure 3A, B?

We sincerely apologize for this confusion. We have corrected it in the revised manuscript (now Figure 3B).

(2) Lines 349-350, "... the invagination speed (represented by the slopes of curves in Figure 5A) gradually slows down at later stages..."

Similar to above, was this meant to be Figure 5C?

Thank you for pointing out this typo. We have corrected it in the revised manuscript (now Figure 5C).

(3) There is a typo in Line 336 ("dimmish") and some minor grammatical concerns in the introduction.

We have corrected the typo "dimmish" to "diminish". We have also carefully proofread the entire manuscript and fixed any remaining grammatical issues.
